# Structure of the (Bi)carbonate Adlayer on Cu(100) Electrodes

**DOI:** 10.1002/anie.202211360

**Published:** 2022-10-13

**Authors:** Reihaneh Amirbeigiarab, Alexander Bagger, Jing Tian, Jan Rossmeisl, Olaf M. Magnussen

**Affiliations:** ^1^ Institute of Experimental and Applied Physics Kiel University 24098 Kiel Germany; ^2^ Center of High Entropy Alloy Catalysis (CHEAC) Department of Chemistry University of Copenhagen Universitetsparken 5 2100 Copenhagen Denmark

**Keywords:** Carbon Dioxide Reduction, Carbonate Adsorption, Density Functional Theory, Electrochemical Interfaces, Scanning Tunnelling Microscopy

## Abstract

(Bi)carbonate adsorption on Cu(100) in 0.1 M KHCO_3_ has been studied by in situ scanning tunneling microscopy. Coexistence of different ordered adlayer phases with ($\sqrt{2}$ ×6$\sqrt{2}$ )R45° and (4×4) unit cells was observed in the double layer potential regime. The adlayer is rather dynamic and undergoes a reversible order‐disorder phase transition at 0 V vs. the reversible hydrogen electrode. Density functional calculations indicate that the adlayer consists of coadsorbed carbonate and water molecules and is strongly stabilized by liquid water in the adjacent electrolyte.

## Graphical abstract

The molecular‐scale structure of the interface between copper electrodes and bicarbonate electrolyte, a key system for electrochemical CO_2_ reduction, is clarified by in situ scanning tunneling microscopy, revealing two highly complex coexisting adlayer structures above a critical potential. Ab initio calculations identify these as carbonate/water coadsorbate layers and indicate a strongly stabilizing effect of the adjacent liquid solution.

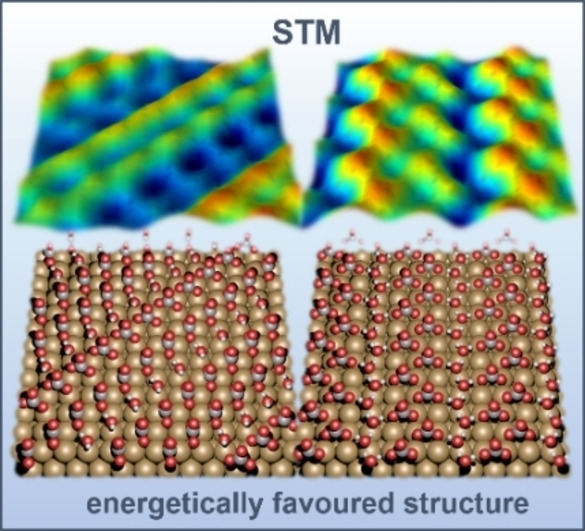

The electrochemical reduction of CO_2_ (CO2RR) on Cu electrodes is a topic of great current interest, as it is a promising route to the sustainable production of valuable feedstock chemicals and fuels.^[1]^ Among the low‐index surfaces, the Cu(100) surface has the highest selectivity for multi‐carbon products and thus has been of particular interest for clarifying on a molecular scale the mechanistic pathways leading to favorable product distributions. For such fundamental understanding, knowledge on the type of adsorbed species on the Cu surface and their adsorption geometry is essential.

The nature of the molecular adlayer of Cu electrodes has been addressed extensively by in situ surface‐enhanced Raman (SERS) and IR absorption spectroscopy, with the main body of work focusing on bicarbonate solutions.^[2]^ The latter are the predominantly employed electrolytes in CO2RR because they provide a buffer with a pH around 7 and can act as a reservoir for CO_2_ via the equilibrium with bicarbonate anions ($\mathrm{HC}O_{3}^{-}$ ). Although bicarbonate is the dominant species in solution, most spectroscopic studies suggest that the adsorbed species on the Cu surface is $CO_{3}^{2-}$ .

Already early FTIRS studies of Hori et al. found a band at ≈1500 cm^−1^ in the double layer range and assigned this to adsorbed $CO_{3}^{2-}$ that was replaced by adsorbed CO in the CO2RR regime.^[2b]^ Subsequent IR spectroscopic studies assigned this band to bidentate carbonate and showed it to decrease below ≈0.1 V vs. the reversible hydrogen electrode (RHE) and to disappear below −0.2 V.[^2c^, ^2d^, ^2e^, ^2f^] Similar observations were made by SERS,[^2g^, ^2h^, ^2i^] although in one of these studies the band was attributed to a carboxyl adsorbate.^[2h]^ In addition, the SERS studies reported a band at 1070 cm^−1^ with a maximal intensity at −0.3 V, which was assigned to monodentate carbonate. In a very recent work by Morazaman and Gul, also an additional weak band at 1344 cm^−1^ was found between −0.2 and 0.4 V, which the authors attributed to adsorbed $\mathrm{HC}O_{3}^{-}$ on the basis of isotope exchange experiments.^[2g]^ In several studies, irreversible changes in the spectra after cycles into the CO2RR regime were reported.[^2d^, ^2i^] All these spectroscopic studies in bicarbonate electrolytes have been performed on polycrystalline Cu, often on roughened or porous samples to make use of surface enhancement effects.

Much less is known about the microscopic structure of the adlayer at the Cu ‐ bicarbonate electrolyte interface. While Cu in acidic and alkaline solutions has been extensively studied by in situ scanning tunneling (STM) and atomic force microscopy (AFM),^[3]^ studies in bicarbonate solution are scarce up to now. Kim et al. observed that polycrystalline Cu reconstructed at −0.9 V vs. the standard hydrogen electrode (SHE) into the (100) face.^[4]^ Simon et al. investigated the Cu surface morphology by in situ AFM measurements.^[5]^ They revealed that immersion at the open circuit potential resulted in a rough surface and that subsequent changes of the potential into the CO2RR range resulted in morphological transformations, in which the number of step sites increased. In addition, a partial coverage by a *p*(2×2) structure was observed on the atomic scale at −0.5 V, which was tentatively attributed to adsorbed CO. No microscopic data on the carbonate adlayer structure has been reported up to now for Cu or any other electrode surface. Only for Ag(110) under ultrahigh vacuum (UHV) conditions, where the carbonate was formed by reaction of CO_2_ with the (1×2)‐O reconstructed surface, a carbonate adlayer with only local order was observed in STM studies.^[6]^

Apart from experimental studies, also ab initio theory has been employed for clarifying the adlayer structure. Density functional theory (DFT) studies of relevant adsorbed species on Cu(100) in CO2RR indicated the presence of carbonate^[7]^ and phosphate^[8]^ anions at low overpotential. Potentially these species block the surface and limit the reaction, which proceeds via. CO reaction species such as the CO dimer (OCCO)^[9]^ or protonated dimer (OCCOH) at slightly higher over‐potentials.

We here present a combined study on the molecular structure of the carbonate adlayer on Cu(100) electrodes in 0.1 M KHCO_3_ by in situ STM and DFT calculations. Coexistence of different, rather complex adlayer structures is observed. On the basis of the DFT results, these can be attributed to the effect of water coadsorption, which stabilizes carbonate on the Cu surface.

The STM experiments were performed on Cu(100) in 0.1 M KHCO_3_ electrolyte, the most common electrolyte for CO2RR, and under argon or CO_2_ gas atmosphere. Experimental details are provided in the Supporting Information. All potentials are given vs. RHE.

Cyclic voltammograms (Figure S1) show only double layer charging without any adsorption peaks between the CO2RR regime and the onset of Cu oxidation above 0.3 V. Nevertheless, we observe in the potential range above 0 V, coexistence of two ordered adlayer phases, one with a stripe‐like (Figure 1a, b) and one with a square appearance (Figure 1c,d). Considering the spectroscopic^[2]^ and ab initio theory[^7^, ^10^] results, we attribute these phases to an adlayer of adsorbed $CO_{3}^{2-}$ or $\mathrm{HC}O_{3}^{-}$ anions. The stripe‐like structure exhibits a long‐range modulation of (2.1±0.1) nm perpendicular to the stripes and an intermolecular distance of (0.35±0.03) nm along the stripes. The stripes are oriented along the [010] and [001] directions of the Cu substrate. These structural parameters are consistent with a ($\sqrt{2}\times$ 6$\sqrt{2}$ )R45° superstructure. High‐resolution STM images reveal a complex molecular arrangement within the adlayer unit cell, consisting of double rows of prominent maxima (Figure 1b), with each second double row appearing slightly higher (Figure S2). Furthermore, additional rows of weaker maxima are visible between the prominent double rows. Along the stripe direction, the maxima in neighboring rows can be aligned or laterally shifted relative to each other.

**Figure 1:**
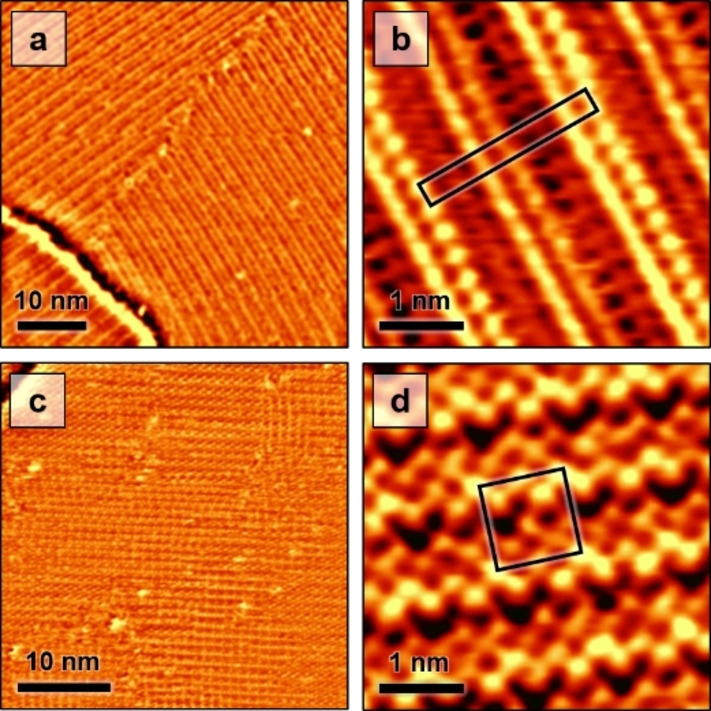
Representative in situ STM images of Cu(100) in 0.1 M KHCO_3_ in the double layer regime. The images were recorded at 0.14 V and show adlayer phases with a, b) a stripe‐like ($\sqrt{2}\times$ 6$\sqrt{2}$ )R45° and c, d) a square (4×4) structure (unit cells indicated in b and d).

The second adlayer phase can be described by a square lattice with a spacing of (1.02±0.05) nm, which is oriented at an angle of 45° relative to the stripes in the ($\sqrt{2}\times$ 6$\sqrt{2}$ )R45° phase, i.e., parallel to the Cu[011] direction. This corresponds to a (4×4) superstructure. The appearance of this (4×4) structure in high‐resolution STM images can be quite variable (see also Figure S3), but the images share certain typically characteristics. Similar to the ($\sqrt{2}\times$ 6$\sqrt{2}$ )R45° phase, also here prominent molecular rows or double rows are observed, with a spacing of 2d_Cu_ between the maxima within each row and an additional weak modulation with a period of 4d_Cu_ (Figure 1d). In between these prominent rows, parallel rows of weaker maxima are found. Adjacent rows are anti‐phase shifted, resulting in a local arrangement of neighboring maxima along the [010] and [001] directions, i.e., in the same direction as in the molecular rows in the ($\sqrt{2}\times$ 6$\sqrt{2}$ )R45° phase.

Both the ($\sqrt{2}\times$ 6$\sqrt{2}$ )R45° and the (4×4) structure, can exhibit different appearances in high‐resolution STM images. These may result partly from the state and shape of the STM tip, which is known to strongly affect the imaging of adsorbed anion adlayers, as e.g. reported for sulfate on Au(111).^[11]^ However, we also observe coexistence of several phases with similar unit cells but different appearance in the same image (Figure S3 and S4), which indicates that different phases with ($\sqrt{2}\times$ 6$\sqrt{2}$ )R45° and (4×4) structure can concurrently exist on the surface. This as well as the parallel presence of adlayer phases with different unit cells suggests some structural flexibility in the adlayer of adsorbed anions that apparently permits formation of a variety of structurally related ordered phases. A coexistence of different adlayer phases, specifically of ordered and disordered structures, was also observed in in situ STM studies sulfate and phosphate adlayers on Cu(111) in neutral solutions.^[12]^

Before discussing the nature and molecular structure of the carbonate adlayer phases in detail, we address the adlayer's dynamics and potential dependency. For the ($\sqrt{2}\times$ 6$\sqrt{2}$ )R45° phase, only weak dynamic fluctuations at fixed potential are observed.

In contrast, the (4×4) phase exhibits strong fluctuations with time. As illustrated in Figure 2a, the local orientation of the characteristic rows of the (4×4) structure changes drastically on the time‐scale of minutes. This can be assigned to positional fluctuations of the boundaries between the 2 different 90° rotational domains of this phase. Such positional fluctuations as well as fluctuations between (4×4) structures of different appearance are also found in high‐resolution images (Figure S3). In agreement with these findings, clear observation of domain boundaries in the (4×4) was difficult, which is attributed to the high boundary mobility. In addition, we observed in images with multiple adlayer domains occasionally areas in which the adlayer appeared disordered (Figure S5 and S6). These may correspond to very small, highly fluctuating domains that cannot be imaged within the time‐resolution of the STM.

**Figure 2:**
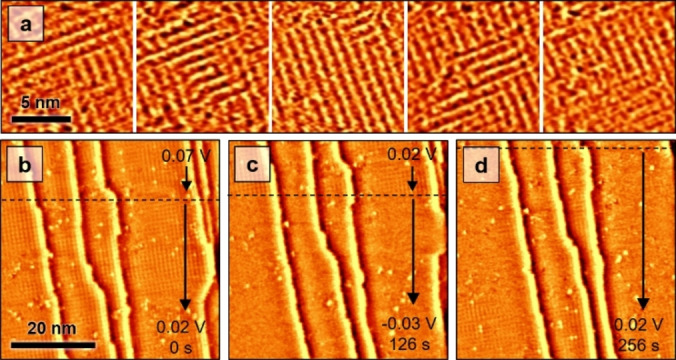
Consecutive sequences of in situ STM images of Cu(100) in 0.1 M KHCO_3_. a) Equilibrium fluctuations in the (4×4) phase at 0.14 V (images recorded at 63 s intervals). Displayed are sections from larger STM images (Figure S5) that are corrected for thermal drift to show the same area. b–d) Sequence recorded during step‐wise potential changes from b) 0.07 to c) 0.02 (upper edge) and then to −0.03 V (dashed line), resulting in disordering of the adlayer. Upon changing the potential back to 0.02 V (d, upper edge), the adlayer slowly reorders again.

At potentials around 0 V, a potential‐dependent order‐disorder transition in the anion adlayer is observed (Figure 2b–d), where the ordered structure immediately disappears upon a potential step to −0.03 V (Figure 2c). After changing the potential back to 0.02 V (Figure 2d), the adsorbate layer starts to slowly reappear on time scales of minutes. This order‐disorder transition was found in the same potential range for the (4×4) (Figure 2b) and the ($\sqrt{2}\times$ 6$\sqrt{2}$ )R45° phase (Figure S7), supporting that both phases are closely related and consist of the same adsorbate species.

The two adlayer phases were found in both Ar and CO_2_ saturated 0.1 M KHCO_3_. Furthermore, no significant changes in the adlayer were observed in STM experiments where the argon gas in the STM chamber was exchanged at about 0.14 V by CO_2_ gas during the experiment. Apparently, the presence of dissolved CO_2_ and the resulting slightly different pH does not have a major effect on the adlayer structure. Furthermore, we do not observe changes in the Cu surface morphology (e.g., in the position of the steps) during the order‐disorder transition in the adlayer (see Figure 2b–d). This indicates that the adlayer formation does not involve surface reconstruction with long‐range mass transport.

Determination of the precise composition and structure of the experimentally observed, rather complex adlayer phases is challenging. For both structures presented in Figure 1, maxima of different apparent heights are observed in the high‐resolution STM images, indicating that adsorbed species are either of different chemical nature or adsorbed on different adsorption sites of the Cu(100) surface. However, the latter alone cannot explain the observations, because some of the maxima differ in height although their lateral positions indicate identical adsorption sites on the Cu lattice. Thus, coadsorption of different chemical species is required to explain the STM results.

Various species may account for such coadsorption. First, carbonate as well as bicarbonate anions exist in this system. Each of those has been proposed as active surface species in the double layer potential regime in previous spectroscopic studies.^[2]^ Most spectroscopy experiments and simulations performed on copper electrodes identify carbonate as the adsorbed species. However, parallel adsorption of $CO_{3}^{2-}$ and $\mathrm{HC}O_{3}^{-}$ is not fully excluded, as indicated by results for other oxyanions. Cyclic voltammetry studies of Gisbert et al. on phosphate adsorption on platinum electrodes indicated that both ${\mathrm{HPO}}_{4}^{-}$ and ${\mathrm{PO}}_{4}^{3-}$ are adsorbed on Pt(111) at neutral pH.^[13]^ It thus may be possible that the adlayer phases in our system consists of coadsorbed $CO_{3}^{2-}$ /$\;\mathrm{HC}O_{3}^{-}$ . However, in this case the ratio of these species should be affected by the pH (i.e., the presence of CO_2_), for which we found no evidence.

Second, $CO_{3}^{2-}$ or$\;\mathrm{HC}O_{3}^{-}$ may be coadsorbed with a water species. The latter has been proposed for ordered adlayers of other oxyanions, such as sulfate and phosphate, on (111) and (100) surfaces of fcc metals in acidic media, where likewise two type of maxima with different apparent height were reported in STM studies.[^11^, ^12^, ^14^] The ordered structures were described in these studies as coadsorption phases of the oxyanions and hydronium ions^[14b]^ or water molecules.^[14c]^ A similar scenario was also proposed for the adsorption of nitrate on Cu(100).^[15]^ Also in neutral electrolyte, an ordered sulfate and phosphate adlayers were reported on Cu(111) and attributed to anion/water coadsorption.^[12]^ Indication for adsorption of oxygen containing species on Cu single crystal surfaces was found in a number of studies.^[16]^ Friebel et al. proposed competitive adsorption of such species and sulfate on Cu(111) at neutral to alkaline pH and suggested that this effect inhibited previously the observation of ordered sulfate and phosphate adlayers in neutral media.^[12a]^

Furthermore, our observations resemble those found for other molecular anions, such as formate and acetate, which have a similar bidentate adsorption geometry on metal surfaces as proposed for carbonate.^[17]^ Parallel chain‐like formate structures on Au(111) have been reported in electrochemical environments, with the spacing between chains decreasing with increasing potential.^[17a]^ For acetate adsorption on Au(111) in electrochemical environments, coexistence of two chain‐like structures was found, one of them dominant and one metastable.^[17b]^ Here again, coadsorbed water was suggested as a stabilizer for the acetate adlayer.

To gain insight into the nature of the adsorbed species and the adlayer structure, we carried out a DFT study of a series of adlayers, consisting of carbonate (CO_3_), bicarbonate (HCO_3_), water (H_2_O), and hydroxyl (OH) at different coverages, on Cu(100) slabs with a (4×4) and with a ($\sqrt{2}\times$ 6$\sqrt{2}$ )R45° unit cell, respectively (details given in the Supporting Information). In total we investigate 192 different initial structures in the two cells (see Table S1–S2). In Figure S8 adlayers of different composition and coverage are analyzed by depicting the lowest energy found for each (note that we do not normalize our energies by carbonate or Cu atoms). We find that structures with rows of carbonate and water are the most energetically stable. The energies of all calculated adlayer configurations are shown in Figure S9–S10. On this basis, we now take a detailed look at the mixed carbonate/ water phases.

For Cu(100) slabs with the (4×4) unit cell the most stable adlayer phases contain 4 CO_3_ and 4 H_2_O in the unit cell (corresponding to a coverage of 1/4 ML CO_3_ and 1/4 ML H_2_O). We display the full set of these structures in Figure S11. Here it is observed that multiple simulations (ID=5, 14, 4, 16, 7, 13, 1, 8, 17, 10) have converged into rather similar low energy structures, which are close to a *c*(2×2) arrangement of CO_3_ and H_2_O molecules that are located in bridge sites. Obviously, these structures differ strongly from those observed in the STM experiments. With increasing adlayer energy, we then find structures where water is detached from the surface (e.g. ID=3, ID=12), which we discard in this analysis. However, at a slightly higher energy of 0.89 eV (ID=2, ID=11) structures are obtained that resemble the experimentally observed adlayers (shown in Figure 3b) and will by further analyzed below.

**Figure 3:**
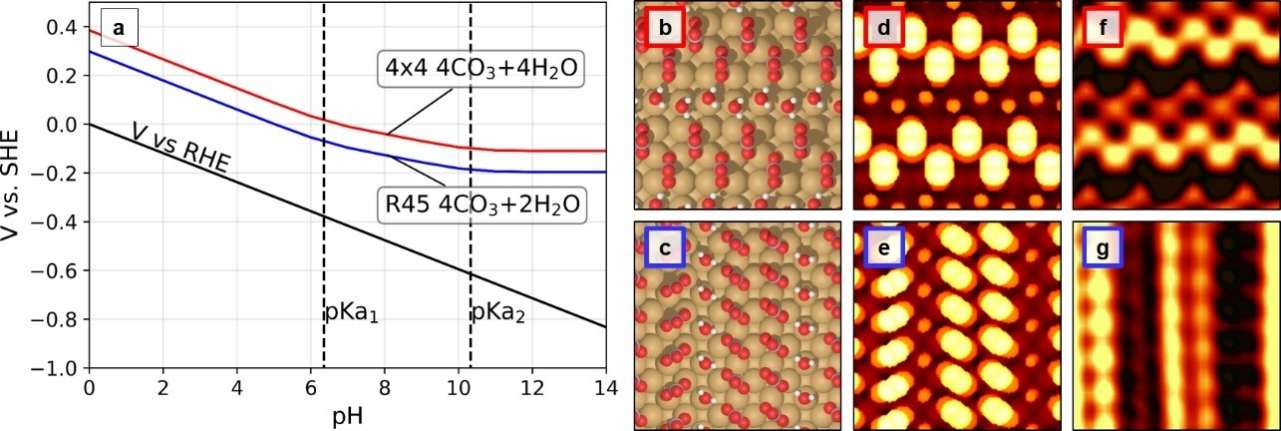
a) Regime of stability of two carbonate and water coverages, (4×4) with 4 CO_3_+4 H_2_O (red) and ($\sqrt{2}\times$ 6$\sqrt{2}$ )R45° with 4 CO_3_+2 H_2_O (blue), in a surface Pourbaix diagram. b, c) Models of the most stable adlayer structures and d, e) corresponding simulated and f, g) measured STM images for each structure.

For Cu(100) slabs with ($\sqrt{2}\times$ 6$\sqrt{2}$ )R45°unit cell the most stable compositions have 4 CO_3_ and 2 H_2_O molecules in the unit cell (i.e., of 1/3 ML CO_3_ and 1/6 ML H_2_O coverage). Here, the energies of the calculated 12 structures with different carbonate and water orientations are within an interval of 0.7 eV (see Figure S12), i.e., differ by <0.18 eV per CO_3_. Because of this small energy difference, positional perturbations and rotations of the carbonate and water molecules are possible, which is in good accordance with the experimental observations of several coexisting adlayer phases. In the lowest energy structure (ID=10), the CO_3_ in the two molecular rows within a CO_3_ stripe are slightly shifted relative to each other; in the slightly less favorable structure ID=2, stripe with this arrangement alternate with stripes where the two CO_3_ rows are anti‐phase shifted. These arrangements already strongly resemble those in the STM images. In the ID=2 structure one of the CO_3_ (the leftmost in Figure S12) is adsorbed with its center almost on top of the Cu and its binding O atoms occupy near‐bridge sites. Moving these O atoms close to on‐top positions results in a structure which resembles more closely the STM images (see Figure 3c) with almost identical energy (3.14 eV), which is chosen for further analysis. We note that this structure, although being low in energy for this composition, it is still about 2 eV higher in energy than our chosen (4×4) structure, which has a 0.08 ML lower CO_3_ coverage. Note that fully optimizing both adsorbates and Cu surface atoms, does not change the structural findings of the carbonate layer on Cu (100) (Figure S13).

In general, the minimum energy structures for the carbonate adlayer obtained in these simulations of Cu(100) in vacuum do not exhibit structural features that correspond well with the experimental data (note that changing functional does not change this conclusion, see Figure S14). This might be caused by missing relevant compositions or structures in the simulations, even for the case of extensive screening as performed here. However, we attribute this failure to another reason, namely the absence of solvating water on top of the carbonate adlayer in the simulations. We have previously observed that carbonate species are strongly stabilized by the aqueous environment, as compared to vacuum simulations by employing ab initio molecular dynamics (AIMD).^[7]^ However, AIMD is computationally not feasible for this screening approach. Instead, we select a subset of the most stable vacuum structures and utilize a continuum solvent method to estimate the effect of water solvation (Figure S15). As expected, these calculations lead to a significant increase in the stability of the carbonate adlayer structures. Moreover, the stabilization induced by the solvent can cause changes in the order of stability of the structures. For example, in the case of the (4×4) unit cell with 4 CO_3_+4 H_2_O the minimum energy structure in solvent is not that found in vacuum (ID=5), but the structure ID=11, which resembles the STM images. Admittedly, the energies are quite close, making both structures possible.

From the continuum solvent simulations we select the two most stable structures within the (4×4) and with a ($\sqrt{2}\times$ 6$\sqrt{2}$ )R45° unit cells and carried out a thermodynamic calculation. The stability is displayed in Figure 3a in a surface Pourbaix diagram (see section 3.4 in the Supporting Information), together with the corresponding structures (Figure 3b, c). The ($\sqrt{2}\times$ 6$\sqrt{2}$ )R45° structure with 4 CO_3_ and 2 H_2_O is slightly more stable than the (4×4) unit cell with 4 CO_3_ and 4 H_2_O, in good agreement with the experimental results. The onset of stability is shifted by about 250 mV towards more positive values in comparison to the experimentally observed order‐disorder transition in the adlayer, which seems acceptable in view of the approximate treatment of the electrolyte solution. According to simulated STM images (Figure 3d, e) of these structures, which are in reasonable agreement with experimental observations (Figure 3f, g), the rows of prominent maxima are formed by carbonate. The rows of molecules with lower apparent height represent adsorbed water molecules, which stabilize the adsorbed anion layer on the electrode surface via hydrogen bridge bonding. Remaining differences to the STM observations may result from relaxations in the Cu surface atoms. In DFT calculations (Figure S13) surface layer relaxations of ≤0.1 Å were found, which is in the range of the minor modulations between the prominent rows assigned to carbonate (Figure S2).

In summary, the combined STM and DFT results provide a first molecular‐scale picture of this important electrochemical interface. They indicate a complex carbonate adsorption behavior where several adlayer structures with comparable stability coexist on the Cu(100) electrode surface. These share certain structural features, especially an arrangement of the CO_3_ adsorbates in stripes with interspersed rows of water molecules and a characteristic CO_3_‐CO_3_ spacing within the stripes that corresponds to the next‐nearest neighbor spacing of the Cu substrate lattice. Our observations suggest that, similar as for other oxyanions, the coadsorption of water plays a key role in stabilizing the adsorbed layer in bicarbonate solution. However, other than in previously studied oxyanion adlayers, sufficient stabilization seems to require not only the presence of coadsorbed water on the Cu surface but also of water in the adjacent molecular layer. Most probably, this next‐layer water also contributes to the stability of the observed structural features, e.g., to the preferred spacing between neighboring CO_3_ via H bridge bonding, but the large number of possible configurations prevents direct testing of this hypothesis by simulations.

According to our observations of a rich and dynamic phase behavior of the carbonate adlayer, the CO_3_‐CO_3_ and CO_3_‐H_2_O interactions are strong enough to stabilize ordered phases while also being sufficiently flexible to allow fluctuations between different structures. It is likely that similar molecular interactions also exist at more negative potentials where the adlayer is disordered. Furthermore, related surface species such as adsorbed CO_2_ and intermediates of the CO_2_ reduction reaction may likewise be ruled by these types of interactions, as suggested by AIMD findings of strong stabilization of such species by water.^[7]^ The ordered carbonate adlayers may thus provide a critical test for the understanding and computational modeling of the CO2RR electrocatalyst interface.

## Conflict of interest

The authors declare no conflict of interest.

## Supporting information

As a service to our authors and readers, this journal provides supporting information supplied by the authors. Such materials are peer reviewed and may be re‐organized for online delivery, but are not copy‐edited or typeset. Technical support issues arising from supporting information (other than missing files) should be addressed to the authors.

Supporting Information

## Data Availability

The data that support the findings of this study are openly available in the repository of the Nano Science Center at the University of Copenhagen at https://nano.ku.dk/english/research/theoretical‐electrocatalysis/katladb/structure‐of‐the‐bicarbonate‐adlayer‐on‐cu100‐electrodes/.
