# Supplementary material for: Structure of the (Bi)carbonate Adlayer on Cu(100) Electrodes
Source: Angew Chem Int Ed Engl. 2022 Oct 13;61(46):e202211360. doi: 10.1002/anie.202211360 (PMC9827965; doi:10.1002/anie.202211360)
Supplement: Supplementary file 1 — Supporting Information [file ANIE-61-0-s001.pdf]

Angewandte Chemie
Eine Zeitschrift der Gesellschaft Deutscher Chemiker

Supporting Information

Structure of the (Bi)carbonate Adlayer on Cu(100) Electrodes

R. Amirbeigiarab, A. Bagger, J. Tian, J. Rossmeisl, O. M. Magnussen**

1. Experimental Section

The experiments were performed in electrochemical environment at room temperature using a PicoPlus STM (Agilent, Santa Clara, USA) and polypropylene-covered tungsten tips. Cu(100) single crystals (MaTecK) were used as working electrode. The copper single crystal was electropolished in 65%- 70% orthophosphoric acid (Merck) at potentials between 1.8 and 2.8 V vs. a Pt wire counter electrode. At least three polishing steps of 10 s each were applied before every experiment. After polishing, the sample was rinsed with ultrapure water, then covered with a droplet of water or 0.1 M H_2SO_4 prepared from ultrapure sulfuric acid (Merck), and finally placed in the STM's electrochemical cell. Two platinum wire were used as pseudo-reference and counter electrodes in the *in situ* STM experiments. Cyclic voltammetry measurements after the experiments and measurement vs. a Standard Calomel Electrode (SCE) were used to calibrate the pseudo reference electrode. In order to avoid oxygen in the electrochemical cell, the electrochemical cell was kept under argon (5.0 N Ar) or CO_2 (4.5 N CO_2) atmosphere during the entire experiment.
The STM data were recorded in constant current mode at tunneling currents of 0.05 nA to 0.7 nA and a bias potential of -0.05 V to -0.35V. The data analysis was carried out with the SPIP software (Image Metrology A/S, Horsholm, Denmark). In some of the images high-pass filtering was applied to increase contrast. Furthermore, in Fig. 1b the average function of SPIP was used to improve the contrast.
The potassium bicarbonate electrolyte was prepared from $KHCO_3$ (Sigma-Aldrich, 99.7%) and ultrapure water and was purified with pretreated Chelex 100 resin (Bio-Rad).[1] Prior to the purification, the Chelex was regenerated in two steps: First, the Chelex was stirred in 1 M HCl for 12 hours and rinsed with 5 l ultrapure water. Then, Chelex was stirred in 1 M KOH for 24 h at about 70 °C and rinsed with 8 l ultrapure water.

2. Supplementary electrochemistry and STM Data

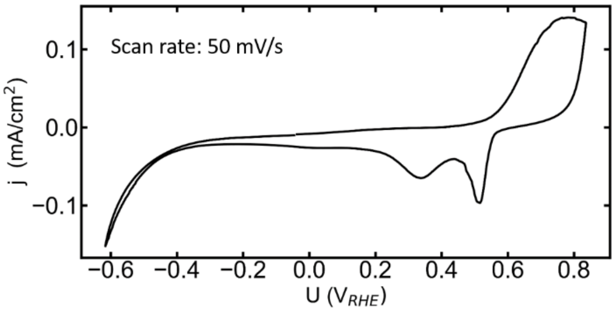

Figure S1. Cyclic voltammetry of Cu(100) in CO_2 saturated 0.1 M $KHCO_3$. The measurement was performed in the electrochemical STM cell.

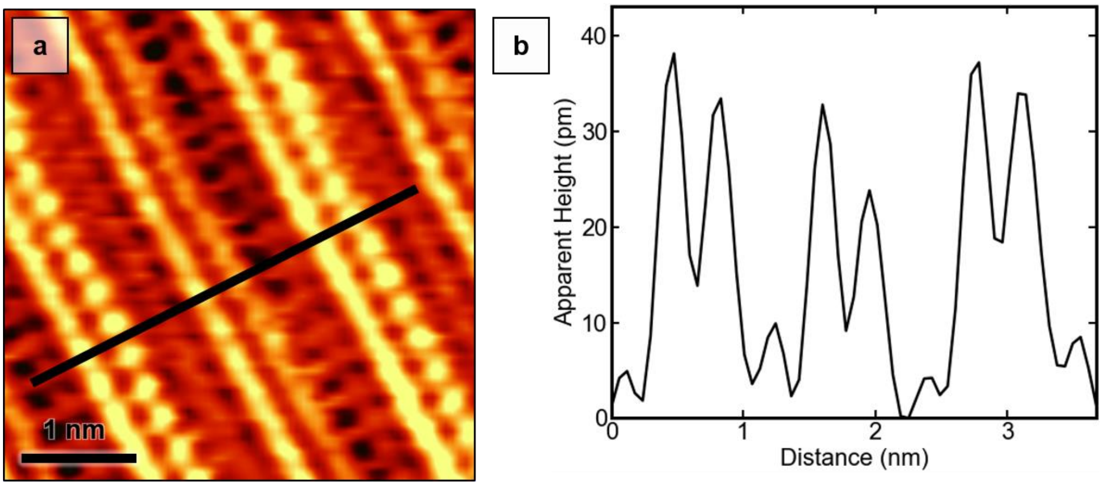

Figure S2. STM data illustrating the alternation in height of neighbouring double stripes in the ($\sqrt{2} \times 6\sqrt{2}$)R45° superstructure. (a) *In situ* STM image of Cu(100) in 0.1 M $KHCO_3$ in the double layer regime (0.14 V). (b) Cross-section taken along black line, showing an apparent height difference of 5-10 pm between neighbouring double stripes, located at distances of 1.1 nm from each other.

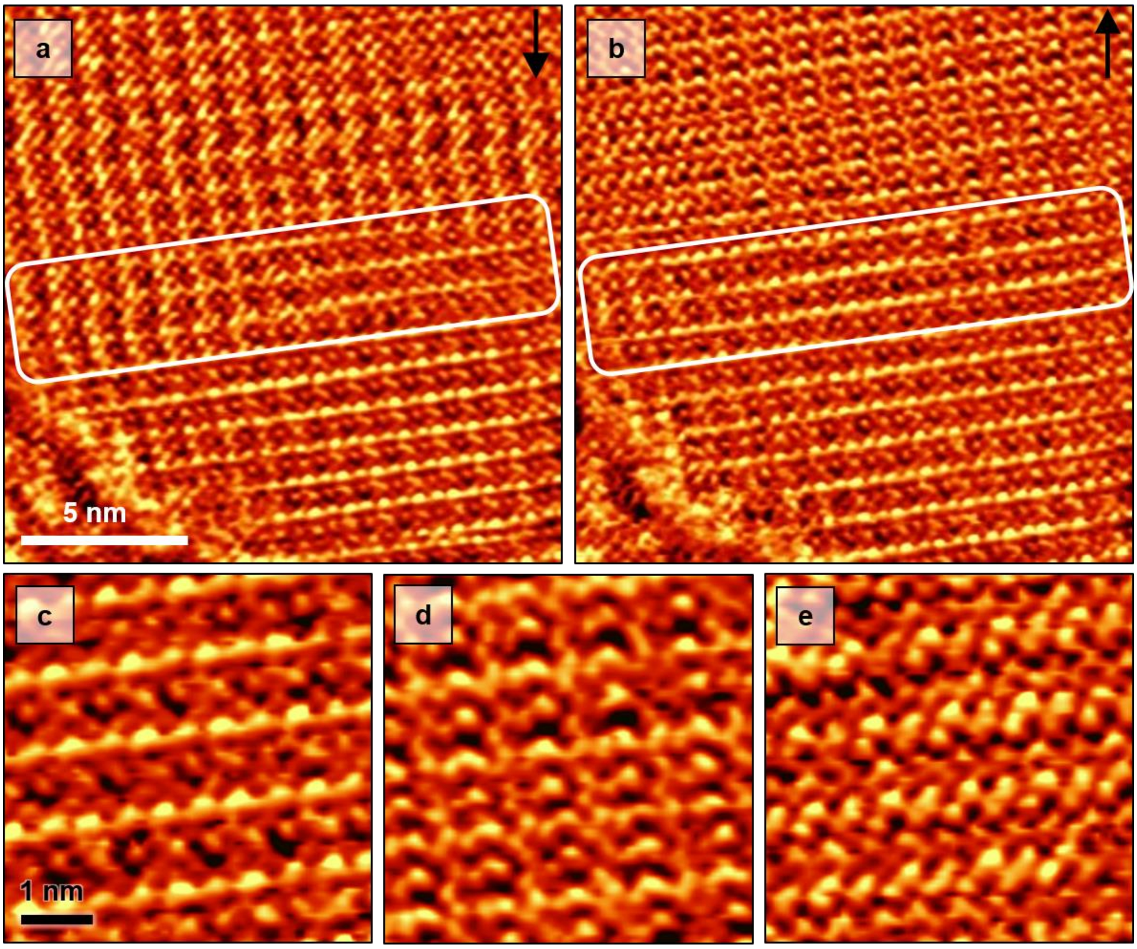

Figure S3. Variable appearance and dynamic fluctuations of (4 × 4) structure. (a) *In situ* STM image taken at 0.14 V, showing coexistence of several (4 × 4) structures with different appearance. (b) Subsequent STM image recorded 85 s later, demonstrating fluctuations within the (4 × 4) structure. Scan directions are marked with arrows. The white rectangular boxes indicate an identical region in both images, in which the (4 × 4) pattern changes to the type of structure visible below the box. (c-e) Sections of the STM image in b, showing (4 × 4) structures with different appearance in more detail.

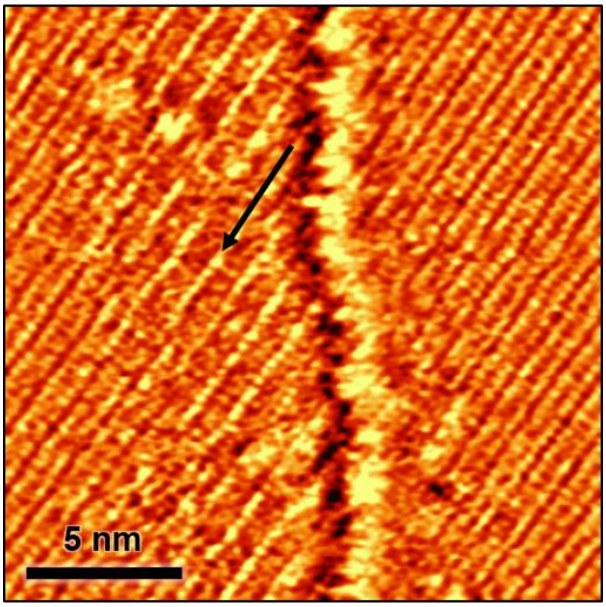

Figure S4. *In situ* STM image of the stripe-like ($\sqrt{2} \times 6\sqrt{2}$)R45° structure in the double layer region (0.14 V, image high-pass filtered for better visibility), illustrating the coexistence of ($\sqrt{2} \times 6\sqrt{2}$)R45° domains with different appearance. Specifically, the appearance along the stripes change from a double row feature to a more prominent single row (marked by black arrow) near a vertical running step in the center of the image.

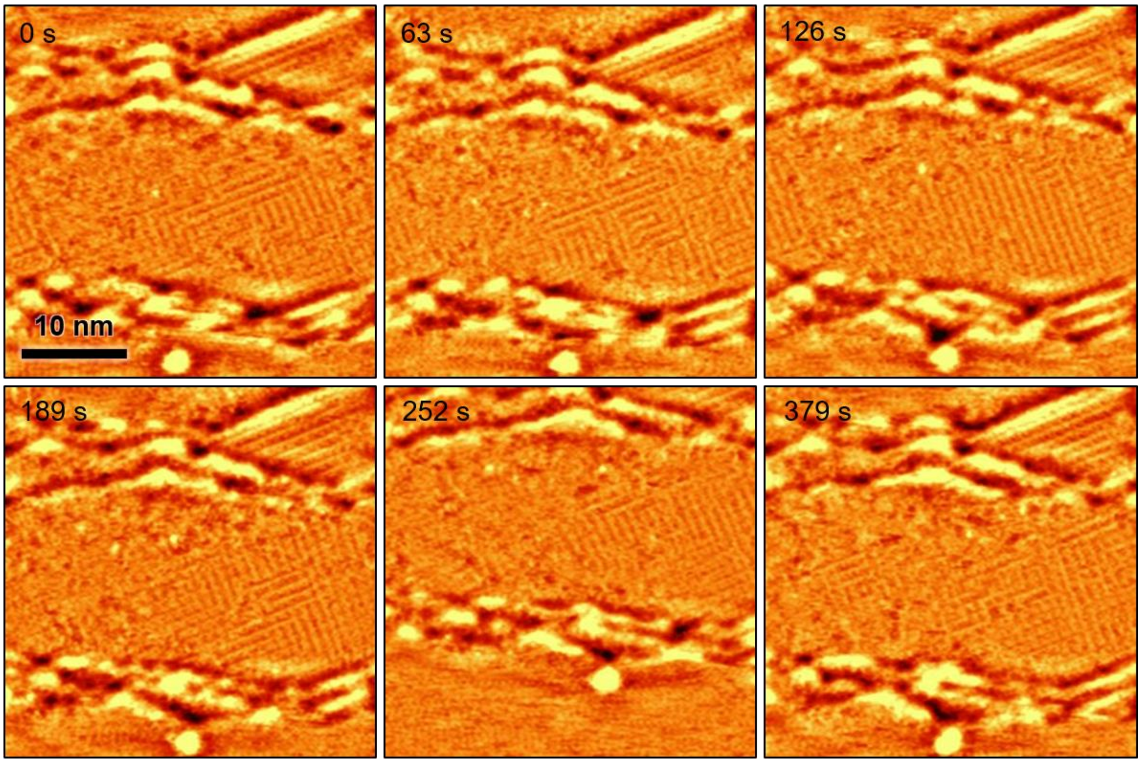

Figure S5. Sequence of *in situ* STM images of Cu(100) in 0.1 M $KHCO_3$ at 0.14 V. All images show the same area on the sample and demonstrate equilibrium fluctuations in the (4 × 4) structure as well as the coexistence of an ordered and an apparent disordered phase. For better visibility of the adlayer structure, the image are high-pass filtered. The Cu steps that terminate the terrace in the center (bright features at the top and bottom edge of the images) serve as markers for identifying identical areas on the sample.

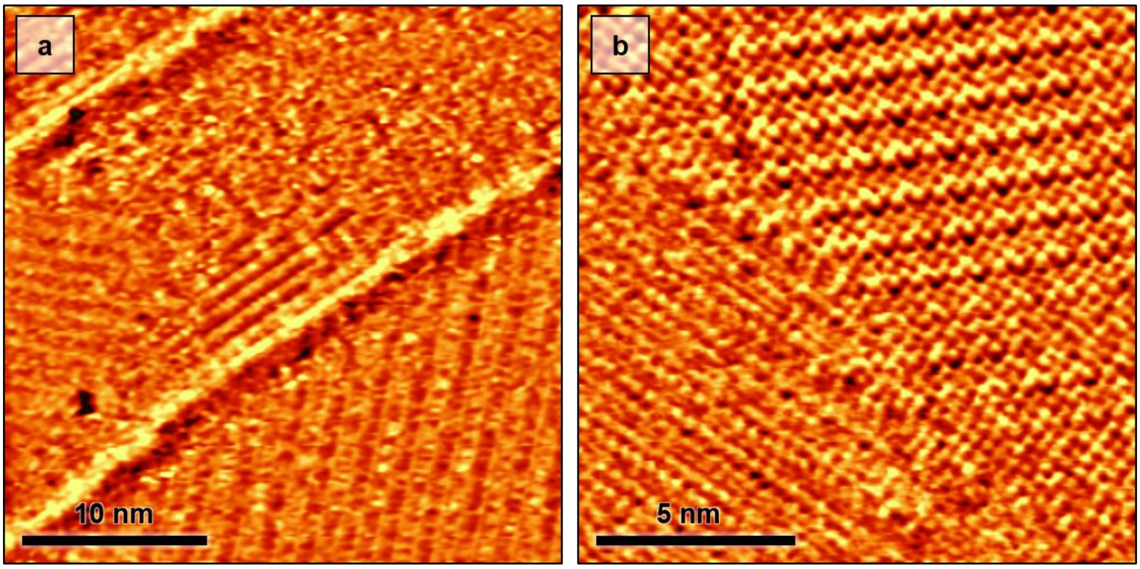

Figure S6. *In situ* STM images of Cu(100) in 0.1 M $KHCO_3$ in the double layer range. (a) Ordered domains with a rotation of about 45° with respect to each other, corresponding to the stripe-like ($\sqrt{2} \times 6\sqrt{2}$)R45° and square (4 × 4) structure, coexist with a disordered phase (0.14 V, image high-pass filtered for better visibility). (b) High-resolution STM image of coexisting domains of the ($\sqrt{2} \times 6\sqrt{2}$)R45° and the (4 × 4) structure (0.14 V).

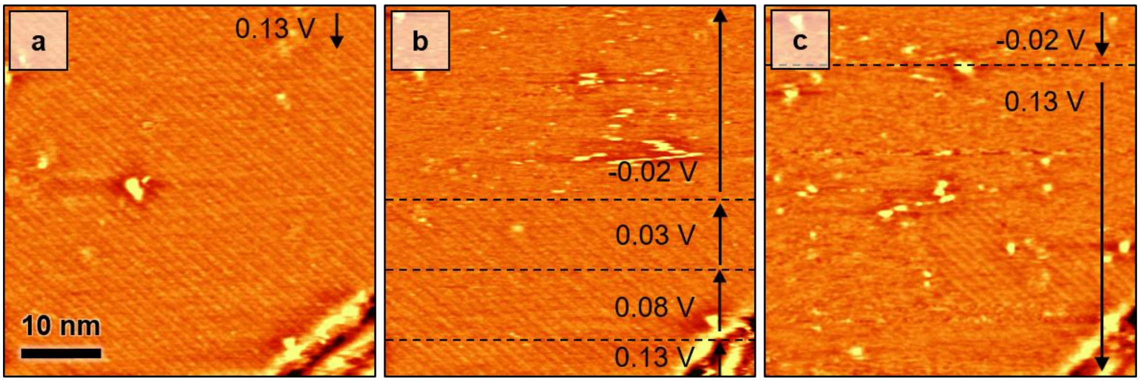

Figure S7. Potential-induced order-disorder transition of the ($\sqrt{2} \times 6\sqrt{2}$)R45° structure. (a-c) Sequential STM images recorded (a) at 0.13 V and (b) during step-wise decrease of the potential (dashed lines) until the ordered structure disappears at -0.02 V. (c) Upon changing the potential back to 0.13 V, the ($\sqrt{2} \times 6\sqrt{2}$)R45° structure slowly start to form again on the electrode surface.

3. Supplementary DFT Data

3.1 Computational details

The simulations were run using ASE [2] with two different metal fcc (100) slabs, a regular (4 × 4 × 3) supercell and a 45° rotated (6√2 × √2 × 3) supercell. In both cases all the Cu layers were fixed to allow a faster force convergence. We test a series of surface compositions (pure CO_3 coverages as well as mixed coverages of CO_3 with HCO_3, OH, and/or H_2O) and several different starting configuration within each composition (see Table S1-S2). Note that in several cases different starting configurations converged into identical adlayer structures during the simulations (e.g., ID=2 and ID=11 in Figure S 10). The binding energies were calculated, using gas-phase CO_2, H_2O and H_2, in the following way (illustrated for pure CO_3 as an example):

$$\Delta E_{*ADS} = E_{*CO3} - E_{CO2(g)} - E_{H2O(g)} + E_{H2(g)} \quad (1)$$

The electronic calculations were carried out at the generalized gradient approximation−density functional theory (GGA-DFT) level of theory, with the projector augmented wave method together with the BEEF-vdW functional [3] as implemented in the GPAW software.[4] A k-point sampling relevant for the specific structure, a grid spacing of 0.18 Å, and a vacuum of 10 Å were applied, and all of the structures were relaxed to a force below 0.05 eV/Å. This means that the binding energies were simulated in vacuum.
To estimate the effect of water solvation we utilize a Continuum Solvent Model (CSM) method as implemented in GPAW [5], with standard parameters for water. Further, to simulate the observed STM images, we utilize the ASE and GPAW STM package.
Structures with total energies, ensembles, and the plotting method are available on the webpage (https://nano.ku.dk/english/research/theoretical-electrocatalysis/katladb/structure-of-the-bicarbonate-adlayer-on-cu100-electrodes/), including the script to plot the data.
We note that because of the high coverage of the adlayer structures, the adsorbate positions can only relax to a limited amount during the structure relaxation. Hence, the structures will be biased by our initial guesses, which we chose on the basis of the experimental observed STM images. For the ($\sqrt{2} \times 6\sqrt{2}$)R45° structures our initial guesses are more similar to each other than for the (4 × 4) structures, resulting in lower energy differences. Specifically, we find for the 4 CO3 + 4 H2O configuration (Figure S11) 3 major energetic groups (Group 1: 0.1 eV, Group 2: 0.8 eV, and Group 3: 1.61 eV) and within each group multiple configurations. Group 1 corresponds to almost square arrangements of the adsorbates, Group 2 corresponds to row-like arrangements of carbonate and of hydrogen bonded water, and Group 3 corresponds to row-like arrangements in which no hydrogen bonds between the water exist.

3.2 Screening coverages

In this section data from the screening approach is shown.

Table S1. Cu(100)-($\sqrt{2} \times 6\sqrt{2}$)R45° structure databases, the coverage and number of structures.

**Cu (100), ($\sqrt{2} \times 6\sqrt{2}$)R45° structure, unit cell (6x2x3)**				
Database name	lowest energy structure	Coverage / composition	Proton-electron transfers	Number of structures
R45_2CO3_2CO3_2H2O.db	ID # 10	4 CO3 + 2 H2O	8	12
R45_2CO3_2CO3_2OH_2H2O.db	ID # 13	4 CO3 + 2 OH + 2 H2O	10	18
R45_2CO3_2CO3_2OH.db	ID # 2	4 CO3 + 2 OH	10	10
R45_2CO3_2CO3_4H2O.db	ID # 15	4 CO3 + 4 H2O	8	30
R45_2CO3_2CO3_4OH.db	ID # 6	4 CO3 + 4 OH	12	14
R45_2CO3_2CO3.db	ID # 1	4 CO3	8	2
R45_4CO3_2HCO3.db	ID # 13	4 CO3 + 2 HCO3	10	31
R45_2CO3_4HCO3.db	ID # 9	2 CO3 + 4 HCO3	12	22
R45_6CO3.db	ID # 2	6 CO3	12	2

Table S2. Cu(100)-(4 × 4) structure databases, the coverage and number of structures.

**Cu (100), (4 × 4) structure, unit cell (4x4x3)**				
Database name	lowest energy structure	Coverage / composition	Proton-electron transfers	Number of structures
Cu4x4x3_4CO3_4HCO3.db	ID # 8	4 CO 3 + 4 HCO3	12	18
Cu4x4x3_6CO3_2HCO3.db	ID # 9	6 CO3 + 2 HCO3	14	12
Cu4x4x3_4CO3_4H2O.db	ID # 5	4 CO3 + 4 H2O	8	18
Cu4x4x3_8CO3.db	ID # 3	8 CO3	16	3

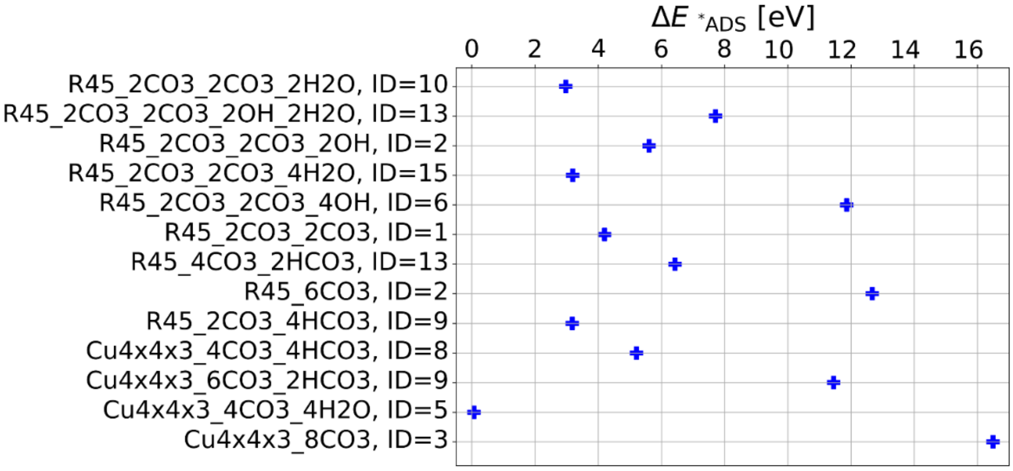

Figure S8. Energetic comparison of all coverages. A general observation is that structures with CO_3 and H_2O are the most stable for both Cu structures.

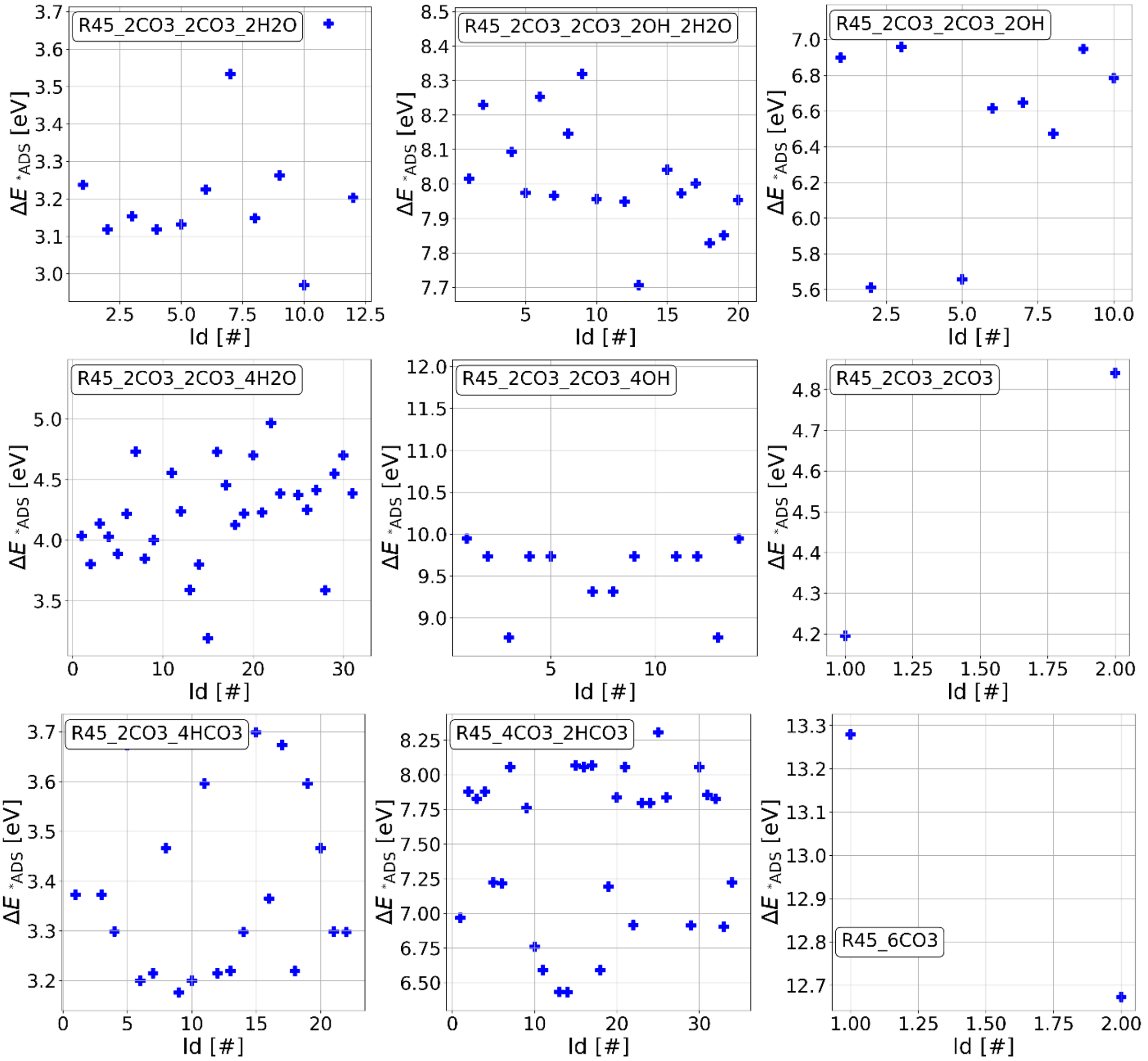

Figure S9. Comparison of ($\sqrt{2} \times 6\sqrt{2}$)R45° structures. Plotted with the energy versus the ID number in the database, and database name written in the plot.

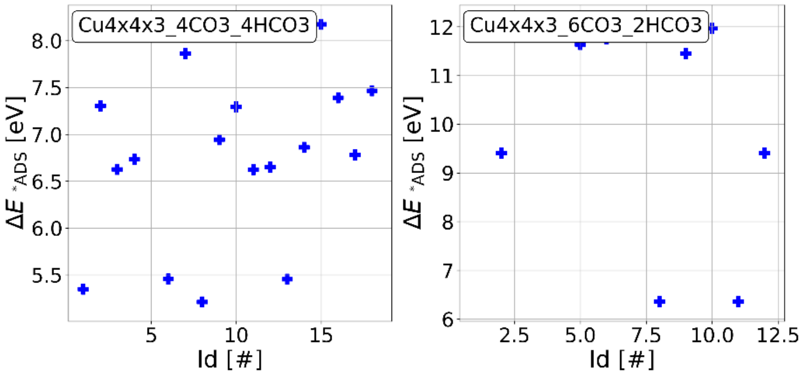

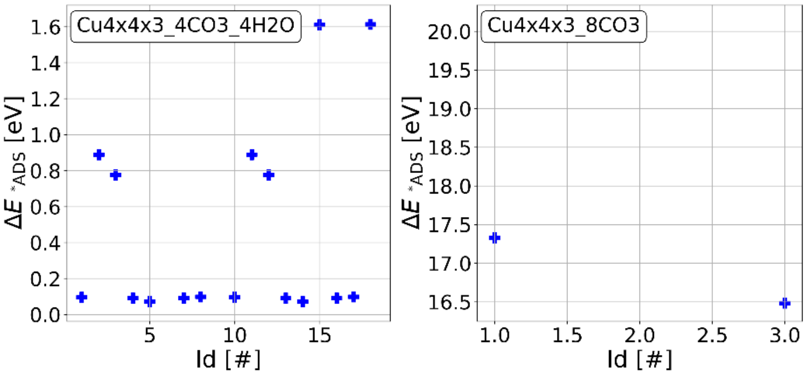

Figure S10. Comparison of structures for (4 × 4) structures. Plotted with the energy versus the ID number in the database, database name is written in the plot.

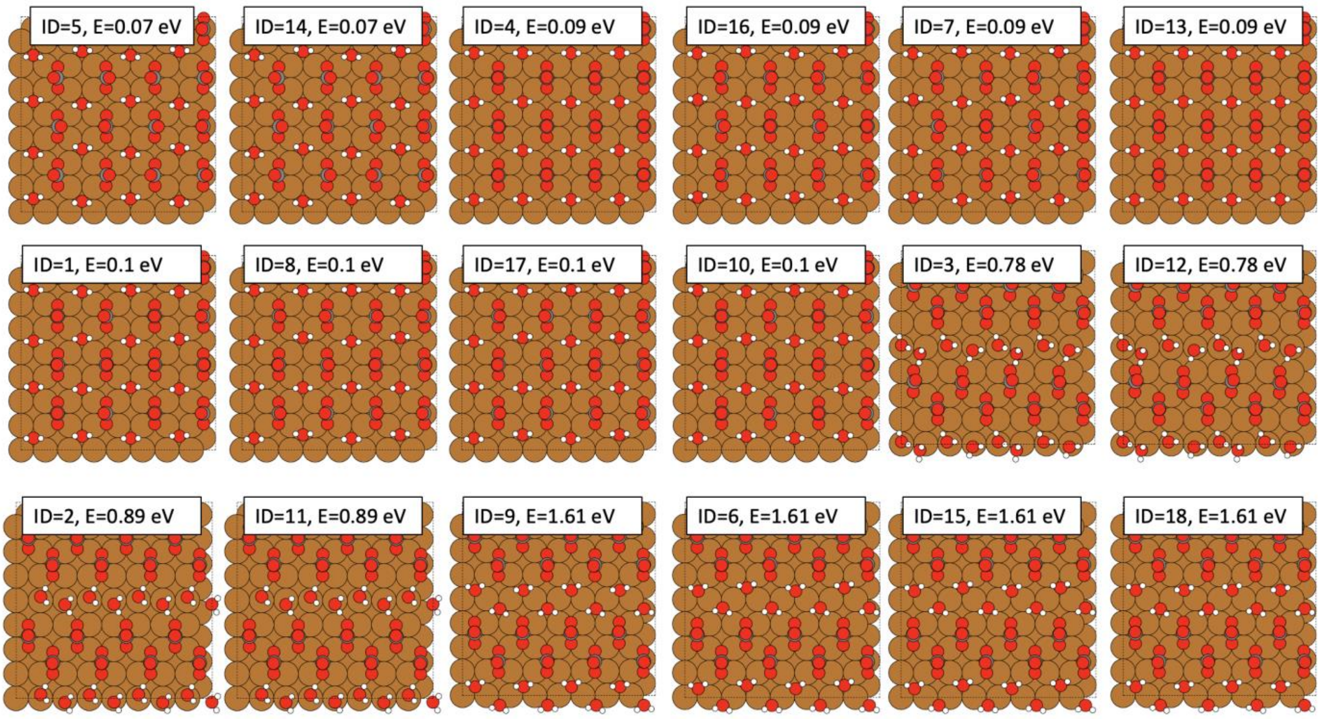

Figure S11. Structural models of lowest energy Cu(100)-(4 × 4) phases, corresponding to a 4 CO_3 + 4 H_2O coverage structures with a visual (2 × 2 × 1) repetition. The structures are sorted with the minimum energy structure first. The data base ID and energy is written in the top box for each structure.

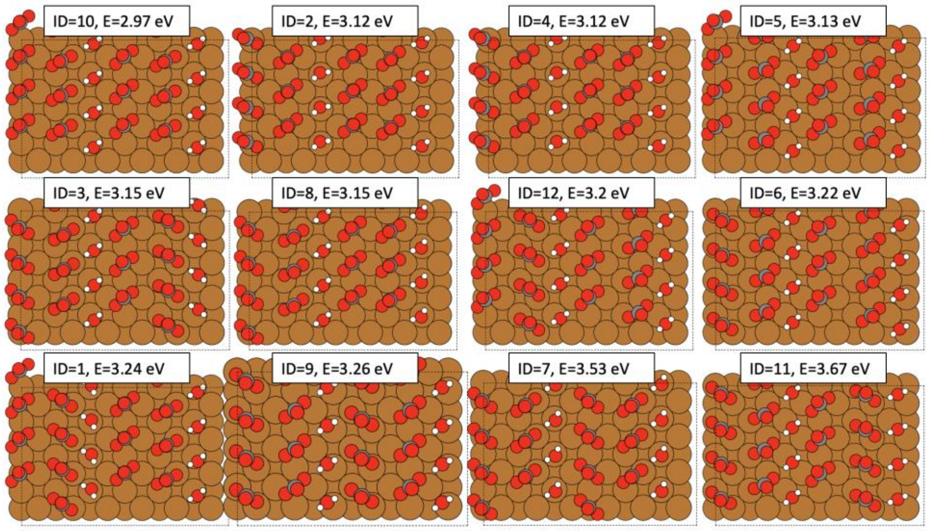

Figure S12. Structural models of lowest energy Cu(100)-($\sqrt{2}$ × 6$\sqrt{2}$)R45° phases, consisting of a 4 CO_3 + 2 H_2O coverage structures with a visual (√2 × 3√2 × 1) repetition. The structures are sorted with the minimum energy structure first. The data base ID and energy is written in the top box for each structure.

3.3 Effect of the employed functional, of Cu substrate relaxation, and of water solvation

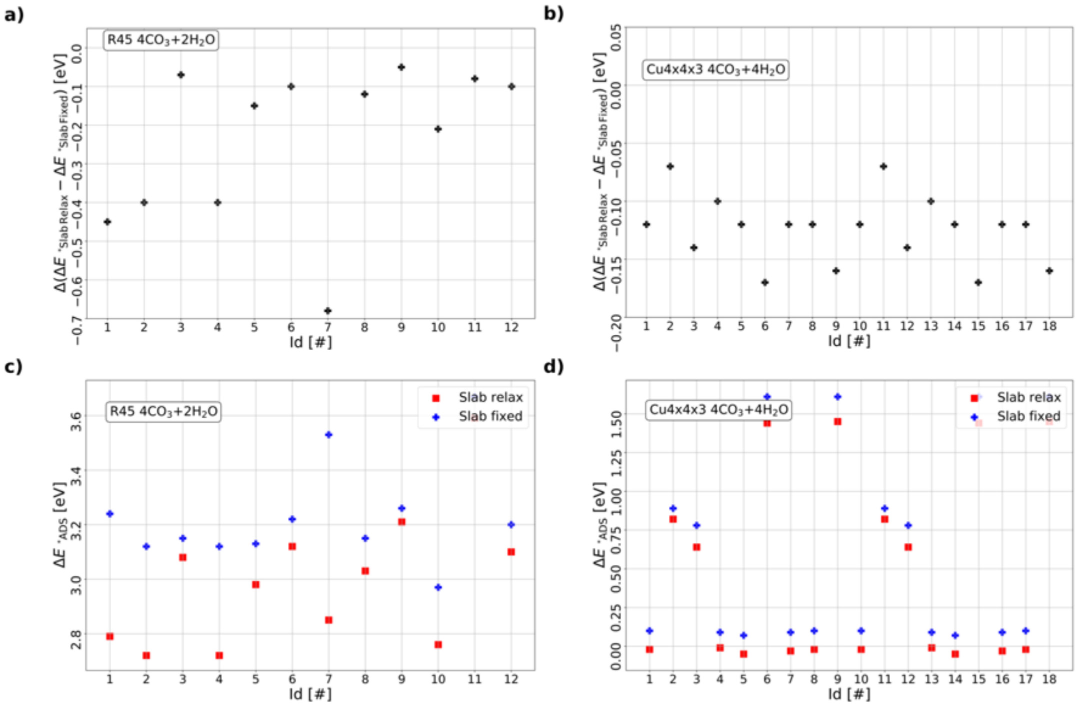

Figure S13. Effect of the relaxation of Cu surface atoms for (a,c) the ($\sqrt{2} \times 6\sqrt{2}$)R45° structure with R45 CO_3 + 2 H_2O composition and (b,d) the (4 × 4) structure with 4 x 4 4 CO_3 + 4 H_2O composition. (a,b) show the energy differences between structures with relaxed and with fixed Cu atoms in the topmost Cu layer, (c,d) show the absolute energies. For the 4 CO_3 + 4 H_2O composition we only observe an almost constant lowering of the energy by about -0.1 to -0.15 eV for all configurations, which is expected when further relaxing a structure. However, for the 4 CO_3 + 2 H_2O composition we find for a couple of configurations a larger drop in energy. This is caused by a reconfiguration of carbonates from on-top/on-top arrangement of the binding oxygen atoms to a more hollow/hollow position. Considering that the final configurations in the main manuscript only have hollow/hollow binding, this seems not problematic.

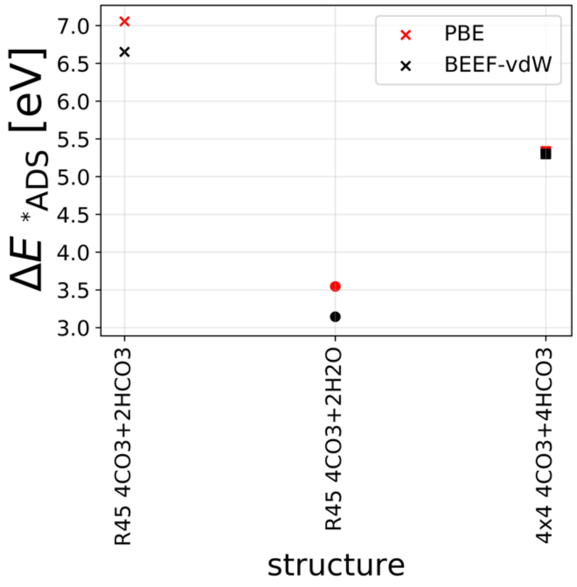

Figure S14. Comparison of simulations employing PBE and BEEF-vdW functionals for three adlayer structures.

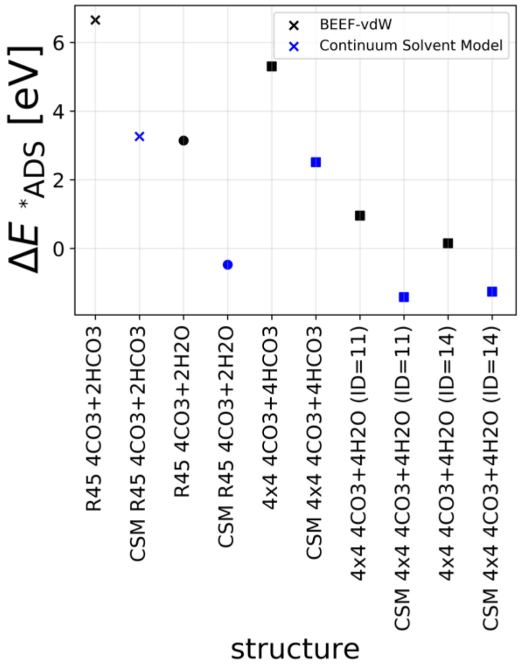

Figure S15. Comparison of simulations of selected adlayer structures in vacuum with those by the Continuum Solvent Method (CSM).

3.4 Pourbaix analysis

In Figure S14 a schematic energy diagram used for the Pourbaix analysis is displayed. The purpose of this approach is to avoid calculating relevant species, such as HCO_3 and CO_3, in solution and instead employ a reference scheme. [6] From this energy diagram the Gibbs free energy of adsorption of carbonate from carbonate species in solution is given by:

$$\Delta G_3 = -\Delta G_1 + \Delta G_2 + n(\varphi - WF - 2.303\ k_b T \times pH) \qquad (2)$$

Here, n is the number of proton-electron transfers, φ is Trassati's value for the absolute electrode potential of -4.4 V vs. SHE, k_b is the Boltzmann constant, T the temperature, pH is the pH, and WF is the work function of the adlayer structure obtained in the simulation. Note that in these vacuum simulations there is only one work function per adlayer structure. In the Pourbaix diagram we are plotting ΔG_3 as a function of the work function, which represents the electrode potential. This is similar to assuming the adlayer energies are not affected by the electrode potential.

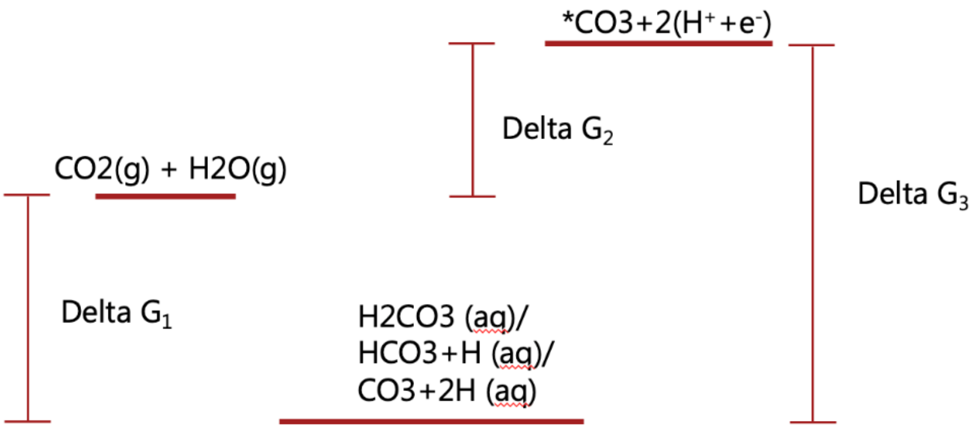

Figure S16. Energy diagram to calculate the stability of adsorbed carbonate ($^*CO_3$) as a function of potential and pH.

Using the equilibrium constants:

$$[HCO_3] = \frac{4.4\times10^{-7}}{[H]+4.4\times10^{-7}},\ \ [CO_3] = \frac{4.69\times10^{-11}}{[H]+4.69\times10^{-11}} \qquad (3)$$

we can now derive the free energy of carbonate species in solution by four different equations as a function of pH:

$$\Delta G(H_2CO_3) = G(H_2CO_3) + K_bT \times \log(1 - [HCO_3]) \quad (4a)$$
$$\Delta G^1(HCO_3 + H) = G(HCO_3) + K_bT \times \log([HCO_3]) + K_bT \times \log([H]) \quad (4b)$$
$$\Delta G^2(HCO_3 + H) = G(HCO_3) + K_bT \times \log(1 - [CO_3]) + K_bT \times \log([H]) \quad (4c)$$
$$\Delta G(CO_3 + 2H) = G(CO_3) + K_bT \times \log([CO_3]) + 2 * K_bT \times \log([H]) \quad (4d)$$

ΔG_1 is now given as a minimum of the four above and the thermodynamic values from Table S3:

$$\Delta G_1 = \min[\Delta G(H_2CO_3), \Delta G^1(HCO_3 + H), \Delta G^2(HCO_3 + H), \Delta G(CO_3 + 2H)] - G(CO_2) - G(H_2O) \quad (5)$$

Table S3. The used Gibbs free energy values at 1 M and pH 0.

Species	CO_2	H_2O	H_2CO_3	HCO_3	CO_3
Gibbs free energy [kJ/mol]	-385.98	-228.57	-623.08	-586.77	-527.81

We now simulated the thermodynamic values of our coverage structure to get ΔG_2:

$$\Delta G_2 = \mathrm{E}_{*ADS} - E_* + ZPE_{*ADS} - \Delta TS_{*ADS} - (E_{molecules} + ZPE_{molecules} - \Delta TS_{molecules}) \quad (6)$$

from which we obtain the Gibbs free energy of adsorption Δ*G₃* via eq. (2).

References

[1] a) A. S. Hall, Y. Yoon, A. Wuttig, Y. Surendranath, *J. Am. Chem. Soc.* **2015**, *137*, 14834-14837; b) J.-J. Velasco-Vélez, T. Jones, D. Gao, E. Carbonio, R. Arrigo, C.-J. Hsu, Y.-C. Huang, C.-L. Dong, J.-M. Chen, J.-F. Lee, P. Strasser, B. Roldan Cuenya, R. Schlögl, A. Knop-Gericke, C.-H. Chuang, *Acs Sustain Chem Eng* **2019**, *7*, 1485-1492.
[2] A. Hjorth Larsen, J. Jørgen Mortensen, J. Blomqvist, I. E. Castelli, R. Christensen, M. Dułak, J. Friis, M. N. Groves, B. Hammer, C. Hargus, E. D. Hermes, P. C. Jennings, P. Bjerre Jensen, J. Kermode, J. R. Kitchin, E. Leonhard Kolsbjerg, J. Kubal, K. Kaasbjerg, S. Lysgaard, J. Bergmann Maronsson, T. Maxson, T. Olsen, L. Pastewka, A. Peterson, C. Rostgaard, J. Schiøtz, O. Schütt, M. Strange, K. S. Thygesen, T. Vegge, L. Vilhelmsen, M. Walter, Z. Zeng, K. W. Jacobsen, *Journal of Physics: Condensed Matter* **2017**, *29*, 273002.
[3] J. Wellendorff, K. T. Lundgaard, A. Møgelhøj, V. Petzold, D. D. Landis, J. K. Nørskov, T. Bligaard, K. W. Jacobsen, *Phys Rev B* **2012**, *85*, 235149.
[4] J. Enkovaara, C. Rostgaard, J. J. Mortensen, J. Chen, M. Dułak, L. Ferrighi, J. Gavnholt, C. Glinsvad, V. Haikola, H. A. Hansen, H. H. Kristoffersen, M. Kuisma, A. H. Larsen, L. Lehtovaara, M. Ljungberg, O. Lopez-Acevedo, P. G. Moses, J. Ojanen, T. Olsen, V. Petzold, N. A. Romero, J. Stausholm-Møller, M. Strange, G. A. Tritsaris, M. Vanin, M. Walter, B. Hammer, H. Häkkinen, G. K. H. Madsen, R. M. Nieminen, J. K. Nørskov, M. Puska, T. T. Rantala, J. Schiøtz, K. S. Thygesen, K. W. Jacobsen, *Journal of Physics: Condensed Matter* **2010**, *22*, 253202.
[5] A. Held, M. Walter, *The Journal of Chemical Physics* **2014**, *141*, 174108.
[6] A. Bagger, L. Arnarson, M. H. Hansen, E. Spohr, J. Rossmeisl, *J. Am. Chem. Soc.* **2019**, *141*, 1506-1514.

Author Contributions

R.A. performed the STM studies including the data analysis, J.T. contributed to the experiments, A.B. performed the DFT calculations, O.M.M. conceived the studies and was responsible for project administration, O.M.M., R.A., and A.B. contributed to writing the original manuscript, and all authors contributed to revising the manuscript.
